# Supplementary material for: Identification, Fine Mapping and Application of Quantitative Trait Loci for Grain Shape Using Single-Segment Substitution Lines in Rice (Oryza sativa L.)
Source: Plants (Basel). 2023 Feb 16;12(4):892. doi: 10.3390/plants12040892 (PMC9966618; doi:10.3390/plants12040892)
Supplement: Supplementary file 1 [file plants-12-00892-s001.zip › plants-2090219-supplementary.pdf]

Supplement

Identification and application of quantitative trait loci for grain shape using single segment substitution lines in rice (*Oryza sativa* L.)

Xiaoling Wang, Xia Li, Xin Luo, Shusheng Tang, Ting Wu, Zhiquan Wang, Zhiqin Peng, Qiyu Xia, Chuanyuan Yu and Yulong Xiao

Supplemental Table S1. Polymorphic markers for SSSLs.

number	mark	number	mark	number	mark	number	mark	number	mark	number	mark	number	mark
1	OSR15	36	PSM306	71	RM16	106	RM242	141	RM330B	176	RM505	211	MM2721
2	OSR22	37	PSM352	72	RM166	107	RM243	142	RM333	177	RM508	212	MM2735
3	OSR30	38	PSM353	73	RM168	108	RM244	143	RM336	178	RM515	213	ID24(4)-2
4	PSM103	39	PSM360	74	RM17	109	RM245	144	RM348	179	RM523	214	ID24(4)-5
5	PSM104	40	PSM363	75	RM18	110	RM246	145	RM3635	180	RM527	215	ID24(4)-6
6	PSM106	41	PSM367	76	RM182	111	RM249	146	RM3794	181	RM535	216	ID31(2)-3
7	PSM125	42	PSM377	77	RM186	112	RM25	147	RM402	182	RM539	217	ID31(2)-5
8	PSM126	43	PSM379	78	RM190	113	RM250	148	RM403	183	RM541	218	ID35(2)-1
9	PSM127	44	PSM380	79	RM19487	114	RM253	149	RM405	184	RM547	219	ID35(2)-3
10	PSM128	45	PSM382	80	RM19516	115	RM257	150	RM407	185	RM549	220	ID40(2)-1
11	PSM132	46	PSM387	81	RM19561	116	RM25718	151	RM410	186	RM55	221	ID40(2)-2
12	PSM136	47	PSM394	82	RM19589	117	RM25753	152	RM411	187	RM556	222	ID42(2)-1
13	PSM137	48	PSM407	83	RM19609	118	RM258	153	RM418	188	RM557	223	ID42(2)-8
14	PSM140	49	PSM455	84	RM19613	119	RM259	154	RM425	189	RM559	224	ID44(2)-1
15	PSM142	50	PSM47	85	RM19637	120	RM264	155	RM430	190	RM562	225	ID48(2)-6
16	PSM143	51	PSM485	86	RM2	121	RM267	156	RM433	191	RM563		
17	PSM145	52	PSM52	87	RM204	122	RM269	157	RM436	192	RM569		
18	PSM152	53	RM10	88	RM205	123	RM271	158	RM437	193	RM571		
19	PSM154	54	RM105	89	RM207	124	RM276	159	RM439	194	RM572		
20	PSM159	55	RM11	90	RM208	125	RM280	160	RM440	195	RM574		
21	PSM16	56	RM1108	91	RM210	126	RM281	161	RM443	196	RM579		
22	PSM161	57	RM111	92	RM214	127	RM282	162	RM446	197	RM580		
23	PSM162	58	RM1186	93	RM216	128	RM283	163	RM447	198	RM581		
24	PSM163	59	RM125	94	RM217	129	RM288	164	RM448	199	RM586		
25	PSM167	60	RM127	95	RM222	130	RM289	165	RM449	200	RM588		
26	PSM169	61	RM128	96	RM223	131	RM293	166	RM468	201	RM591		
27	PSM17	62	RM129	97	RM225	132	RM294A	167	RM474	202	RM592		
28	PSM192	63	RM131	98	RM227	133	RM301	168	RM478	203	RM594		
29	PSM193	64	RM133	99	RM228	134	RM304	169	RM481	204	RM596		
30	PSM202	65	RM135	100	RM23	135	RM306	170	RM490	205	RM633		
31	PSM21	66	RM138	101	RM23158	136	RM308	171	RM493	206	RM6773		
32	PSM283	67	RM146	102	RM234	137	RM311	172	RM5	207	RM70		

33	PSM30	68	RM147	103	RM235	138	RM312	173	RM50	208	RM72
34	PSM304	69	RM149	104	RM237	139	RM329	174	RM502	209	RM80
35	PSM305	70	RM152	105	RM24	140	RM330A	175	RM503	210	RM9

Supplemental Table S2. Polymorphic marker information of insertion and deletion for SSSLs.

Code	Type	Sequence	bp	Production	Code	Type	Sequence	bp	Production
24(4)-2R	Indel	CACACTGGATCCTGGACAGACG	22	294	40(2)-1R	Indel	TATGCTTGTGATAACTCT	18	158
24(4)-2F		CAGCACACACCTCCGTGAAGC	21		40(2)-1F		TATGCTTCTACTTTCTTG	18	
24(4)-5R	Indel	CGGCGCTTGATGGTGATTAGC	21	86	40(2)-2R	Indel	AGACCTTGCTCTGATACC	18	109
24(4)-5F		AGCGCGCCGAGAAGAATAGC	20		40(2)-2F		CATCATTCCAATATGCTA	18	
24(4)-6R	Indel	AACCCATCCATCCAGTCAAAGC	22	126	42(2)-1R	Indel	GTGGCAGTTACTAATACGG	19	130
24(4)-6F		CACCACCGCCTTCAGGTACG	20		42(2)-1F		CTGTGGATAAGGCAGACC	18	
31(2)-3R	Indel	ACTCCATCCAGCATACAC	18	164	42(2)-5R	Indel	GTAGGCTATACCTGACAA	18	132
31(2)-3F		GATTACGTCGGACCAAAA	18		42(2)-5F		TTTATACCTACCAAGTTT	18	
31(2)-5R	Indel	GGTGAGACAGCCAGATTGG	19	126	44(2)-1R	Indel	CTCTACTCGGATTGGTGG	18	115
31(2)-5F		GCTCTGCTCCACCTCCAT	18		44(2)-1F		GATAGTGGTAGCCAGGGA	18	
35(2)-1R	Indel	GTTGCCGTTGCCGTTGCT	18	160	48(2)-6R	Indel	TTCTCATGCCTTCTCCTT	18	169
35(2)-1F		TGCTTGAGCTGGTCGGAGGC	20		48(2)-6F		CTGCATAGACGCCCACAT	18	
35(2)-3R	Indel	TTTCTGTCTCACGCTGGAT	19	102					
35(2)-3F		ACGATGAGCAGTGGTGGA	18						

Supplemental Table S3. TRAP-Seq expression primer.

Gene name	Primer direction	Primer sequence
LOC_Os10g37850.2	Forward	CGAACGCCAGGGACATCAAG
	Reverse	AGCGTGAGGAGGCAGGAGAT
LOC_Os10g37860	Forward	GTTGCCACACTACCATACAT
	Reverse	GGAAGTCTACCAACCGAGGA
LOC_Os10g37870	Forward	GACATGCTCAGCCGATTCTC
	Reverse	CGAGCTGAATTAGCATCAGCAA
LOC_Os10g37880	Forward	CACATTGAAGGGGCCCTGAT
	Reverse	GCCCTCAATCGGTCTTCCAA

Supplemental Table S4. TRAP-Seq FPKM expression values.

Plant tissue	Expression pattern	Candidate genes			
		LOC_Os10g37850.2	*LOC_Os10g37860*	*LOC_Os10g37870*	*LOC_Os10g37880*
Seedling	RNA-Seq FPKM	2.52337	19.8396	9.96971	23.1046
	TRAP-Seq FPKM	3.9476	55.8318	18.3617	9.85228
Callus	RNA-Seq FPKM	2.08115	10.9511	10.8666	27.9305
	TRAP-Seq FPKM	3.13333	71.1632	33.2764	21.4554
Panicles	RNA-Seq FPKM	22.3789	671.641	76.7341	12.3074
	TRAP-Seq FPKM	13.1439	66.5442	17.2411	12.4649

Supplemental Table S5. Substitute fragment information of the biological materials of 33 SSSLs.

Material	Donor	Chr	Substitution segment
S1	IR64	1	RM329--RM24-RM129-PSM367-RM446--RM9
S2	IR64	1	RM572--RM23-RM312-RM493-RM562--RM449
S3	IR64	5	PSM202--RM405-RM267-RM592-RM574--RM437
S4	IR64	10	PSM162--PSM163-RM269-RM304-PSM167-PSM168-RM294A-RM228--RM591
S6	IR64	10	RM216--RM244-RM311-PSM162--RM269
S8	Lemont	1	RM5--RM246-RM443--RM128
S9	Lemont	2	RM425--RM250-RM166-RM208-RM207-RM138-PSM125-RM535—End
S10	Lemont	3	End---RM301-PSM306-PSM305-PSM304-RM523--RM569
S14	Lemont	4	PSM104--PSM103-PSM360-PSM382-RM127-RM280-RM559--End
S16	Lemont	6	RM217--RM253-RM402--RM557
S17	Lemont	7	PSM353---RM505-RM234-RM18---RM478
S18	Lemont	8	PSM394--RM515-RM223-RM556-RM210--RM80
S19	Lemont	8	RM308--RM447-RM264--RM281
S20	Lemont	8	RM502--RM447-PSM352-RM264--RM281
S23	Lemont	10	End--RM474-RM330A-RM222-RM216-RM311-PSM162-PSM163--RM596
S24	Lemont	10	RM271/PSM455--RM258-PSM169-RM147--PSM407
S25	Lemont	8	RM223—RM556-RM210-RM80-RM149--RM433
S27	Zihui100	7	PSM142--RM2-RM214-RM418-RM11-OSR22-RM182-RM336-RM10--RM70
S28	Zihui100	4	PSM106--PSM103-RM348-OSR15-PSM360---PSM382
S29	Zihui100	3	RM293--RM468-RM571--RM227
S30	Zihui100	4	RM348--OSR15-PSM382-RM131--RM127
S31	Zihui100	12	PSM192--RM17--PSM193

S35	Zihui100	7	RM481--PSM142-RM2-RM214-RM418-RM11-OSR22--RM182
S40	IR64	3	PSM126--RM16-RM633-PSM127-PSM380--RM503
S42	Basmati 370	1	RM283--RM259-RM579-RM580-RM572-RM23-RM312--RM562
S43	Lianjian33	1	RM306--RM237-RM246--RM403
S44	Lianjian33	1	RM490--RM259-RM243-RM580-RM572-RM23-RM493--RM562
S45	Lianjian33	1	RM579--RM581-RM580-RM572-RM23-RM312-RM493-RM449--RM594
S46	Ganxiangnuo	3	PSM16--PSM30-PSM21-PSM47-PSM377-PSM379-RM411-RM16-PSM127--PSM128
S47	Kyeema	7	PSM140--RM436-RM481-PSM143-PSM145--RM125
S48	Chenglongshuijin	3	RM135--RM168－RM186-PSM52-RM55-PSM132--RM448
S50	Lemont	6	PSM387--RM133–RM508-RM586-RM588–RM190–RM204–RM253-RM50–RM402--RM557
S51	Lemont	6	End-RM508-RM190–RM204–RM225–RM217–RM402–RM527–RM541-PSM136--PSM137
